# The synbiotic mixture of lactulose and* Bacillus coagulans* protects intestinal barrier dysfunction and apoptosis in weaned piglets challenged with lipopolysaccharide

**DOI:** 10.1186/s40104-023-00882-9

**Published:** 2023-06-11

**Authors:** Weijiang Zheng, Zuyan Zhao, Yunnan Yang, Liren Ding, Wen Yao

**Affiliations:** 1 College of Animal Science and Technology, Nanjing Agricultural University, Nanjing, 210095 Jiangsu China; 2 National Experimental Teaching Center for Animal Science, College of Animal Science and Technology, Nanjing Agricultural University, Nanjing, 210095 Jiangsu China; 3 Key Lab of Animal Physiology and Biochemistry, Ministry of Agriculture, Nanjing, 210095 Jiangsu China

**Keywords:** Apoptosis, *Bacillus coagulans*, Intestinal barrier function, Lactulose, LPS, Piglets, Synbiotic

## Abstract

**Background:**

Lactulose as an effective prebiotic protects intestinal mucosal injury. *Bacillus coagulans* is widely used in feed additives because of its ability to promote intestinal health. Our previous study suggests that the combination of lactulose and *Bacillus coagulans* may be a good candidate as alternative for antibiotic growth promoters. However, the in vivo effects of lactulose and *Bacillus* *coagulans* on growth and intestinal health under immune challenge in piglets remains unclear. The objective of this study is to explore the protective effects of synbiotic containing lactulose and *Bacillus coagulans* on the intestinal mucosal injury and barrier dysfunction under immune challenge in weaned piglets.

**Methods:**

Twenty four weaned piglets were assigned to 4 groups. Piglets in the CON_-saline_ and LPS_-LPS_ group were fed the basal diet, while others were fed either with chlortetracycline (CTC) or synbiotic mixture of lactulose and *Bacillus coagulans* for 32 d before injection of saline or lipopolysaccharide (LPS). Piglets were sacrificed 4 h after LPS injection to collect samples to determine intestinal morphology, integrity and barrier functions as well as relative genes and proteins.

**Results:**

Our data showed that no differences were observed in the growth performance of the four test groups. LPS injection induced higher serum diamine oxidase activities, *D*-lactic acid levels, and endotoxin status, lower villus height and ratio of villus height to crypt depth, greater mRNA and lower protein expression related tight junction in both jejunum and ileum. In addition, a higher apoptosis index, and protein expression of Bax and caspase-3 were also observed in the LPS challenge group. Interestingly, dietary synbiotic mixture with lactulose and *Bacillus coagulans* protected against LPS-induced intestinal damage, barrier dysfunction and higher apoptosis as well as CTC.

**Conclusions:**

Our data suggest that dietary supplementation of synbiotic mixture with lactulose and *Bacillus coagulans* showed resilience to LPS-induced intestinal morphological damage, barrier dysfunction and aggressive apoptosis in piglets as well as the protective effects of CTC. These results indicate that synbiotic mixture of lactulose and *Bacillus coagulans* showed beneficial effects on performance and resilience to acute immune stress in weaned piglets.

**Supplementary Information:**

The online version contains supplementary material available at 10.1186/s40104-023-00882-9.

## Background

The gastrointestinal (GI) tract is not only a place for digestion and absorption of nutrients, but also acts as a barrier to separate the antigens, microorganisms and xenobiotics from the environment into the body [1]. The intestinal epithelium contains crypts and villi, which are composed of single layer of tightly bound columnar intestinal cells that are joined together by junctional complex [2]. Tight junctions (TJs) as the most apical intercellular complex, consisting of TJ proteins claudins, zonula occludins (ZOs), occludin, and junctional adhesion molecules (JAMs), which controls the permeability of the paracellular transport pathway [3]. The integrity of the gut barrier function is critical to the growth, health and welfare of animals. There is considerable scientific evidence that bacterial infection, endotoxin challenge, may impair barrier function and small intestine integrity in animals [4, 5]. Indeed, piglets with immature intestines are commonly under weaning stress, including physiological, environmental and social stressors on modern swine farms [6]. These stress factors during weaning make it easier for piglets to develop intestinal mucous epithelium disturbance, diarrhea, and even death, resulting in severe economic losses to the pig industry [7]. Thus, the subtherapeutic level of antibiotics was used as growth promoters to improve the health and growth performance of swine during and after weaning stage [8]. However, it has been reported that long-term prophylactic use of antibiotic in pigs can cause more severe problems such as antibiotic resistance bacteria and environmental pollution [9]. Furthermore, antimicrobial growth promoters (AGPs) in animal feed have been banned in Europe, Australia and China [10]. Consequently, more and more studies have been devoted to the search for alternatives to antibiotic growth promoter, including probiotic, prebiotics and synbiotic.

As a kind of probiotic, *Bacillus coagulans* is a Gram-positive lactic acid-producing bacterial species and its spores have strong resistance to acid, high temperature, high pressure, and easy storage properties [11]. Earlier evidence has indicated that *Bacillus coagulans* could improve the growth performance and intestinal health in animals [12, 13]. Lactulose is a prebiotic made up of galactose and fructose which cannot be metabolized by the digestive enzymes in animals and therefore directly fermented by the microbe in the gastrointestinal tract [14]. It is well documented that dietary lactulose supplementation would beneficial for the growth performance and intestinal health of pigs [15, 16]. In addition, our previous data indicated that the combination of lactulose and *Bacillus coagulans* has more potential beneficial effects on the microbiota metabolism of the microbiota and its by-products [17]. Based on the positive effects of *Bacillus coagulans* and lactulose, we hypothesized that the synbiotic mixture of lactulose and *Bacillus coagulans* may be a good potential alternative for in-feed antibiotic growth promoter. Lipopolysaccharide (LPS) is an endotoxin from the cell wall of Gram-negative bacteria, has been proven causing various morphologic response in the intestine, including villus atrophy, epithelial vacuolation, barrier dysfunction and apoptosis[18]. Previous studies have shown that LPS can be used as a well-established model of intestinal injury [19]. So, the objective this study was to investigate whether the synbiotic mixture of lactulose with *Bacillus coagulans* as alternative of CTC could protect the dysfunction of intestinal damage, barrier dysfunction and apoptosis in weaned piglets challenged with LPS.

## Methods

### Animals and experimental design

The experimental design and procedures in this study were approved by the Animal Care and Use Committee of Nanjing Agricultural University (Approve number 20190819), Nanjing, China. Twenty four clinically healthy castrated male weaned piglets [Duroc × (Yorkshire × Landrace), initial body weight of 9.09 ± 0.12 kg] from 6 litters (27–28 days of age, 4 pigs/litter, sow parity at 3) were individually kept in pens (1.2 m × 2.1 m). Each pen was equipped with a plastic floor, feeder and nipple drinker to provide ad libitum access to feed and water. Piglets from one litter were equally assigned to one of the 4 groups according to their BW: negative control (CON), positive control (LPS), chlortetracycline (CTC) and synbiotic (SYN) groups, respectively. This trial consisted of an 8-d adaption period and a 33-d (from d 0 to 32) experimental period, respectively. The experimental design schematic is presented in Additional file 1: Fig. S1. A corn-soybean meal-wheat bran-fish meal-based antibiotic-free diet was formulated to meet the nutrient requirements of the National Research Council (NRC, 2012) for 7–11 kg and 11–25 kg stage [20]. The compositions of the diet are shown in Additional file 1: Table S1. Piglets in the CON and LPS groups were fed the basal diet. Piglets in the CTC group received basal diet with 75 mg/kg chlortetracycline (CTC; Wellhope Foods Co., Ltd., Shanghai, China), while the SYN group were fed basal diet with synbiotic mixture of 10 g/kg lactulose (Abbott Healthcare Products, Weesp, The Netherlands) and 2 × 10^9^ CFU/kg *Bacillus coagulans* (Jiangsu Yuanshan Biological Technology Co., Ltd., Yancheng, China).

Weekly body weight, daily food intake, illness and any abnormal behavior were recorded. Piglets were closely observed daily for clinical signs of diarrhea as previously described [21, 22]. After treating with different diets for 28 d, fasting blood sample was collected from the anterior vena cava of each piglet and following a 3-d recovery period. At the end of the experiment, piglets were fasted overnight (12 h) before and after the saline or LPS challenge. Those piglets in LPS, CTC and SYN groups (as group ID changed to LPS_-LPS_, CTC_-LPS_ and SYN_-LPS_, respectively) were challenged intraperitoneally with LPS (*E. coli* serotype O55:B5, Sigma Chemical Inc.) at 100 μg/kg BW dissolved in sterile saline, while the pigs in the CON group (as group ID changed to CON_-saline_) were given an equivalent amount of sterile saline [19]. The LPS dose was used in accordance with previous studies [23, 24]. As soon as saline or LPS injection, piglets were orally given *D*-xylose at the dose of 500 mg/kg BW prepared as 0.5 g/mL solution in deionized water according with previous study [25].

### Sample collection

At 4 h after saline or LPS challenge, a 5-mL of blood sample was collected from the jugular vein of each piglet, and after being placed on ice for 30 min followed by centrifugation at 3,000 × *g* for 20 min at 4 °C to obtain serum. The serum samples were then stored in a pyrogen-free glass tube (Chinese Horseshoe Crab Reagent Manufactory, Xiamen, China) at −80 °C. Then, piglets were euthanized by an intramuscular injection of sodium pentobarbital (40 mg/kg BW) and the small intestine was rinsed with ice-code physiological saline. The jejunum (6 cm before the end of jejunal Peyer’s patches, 2 cm) and ileum (6 cm from the ileal-cecal junction) segments were cut and fixed in 4% paraformaldehyde or 2.5% glutaraldehyde for morphological analysis. Mucosa samples of duodenum, jejunum, and ileum were scraped and immediately snap-frozen in liquid nitrogen and stored at −80 °C for RNA and protein extraction.

### Serum intestinal barrier function biomarkers

Serum intestinal barrier function biomarkers, including enzymatic activity of intestinal diamine oxidase (DAO), the concentrations of *D*-xylose and *D*-lactic acid were measured by enzymatic spectrophotometry using a commercial kit (Nanjng Jiancheng Bioengineering Institute, Nanjing, Jiangsu, China). Free LPS in the serum was measured by chromogenic end-point Tachypleus Amebocyte Lysate assay kit (Chinese Horseshoe Crab Reagent Manufactory, Xiamen, China) with a minimum detection limit of 0.01 endotoxin units (EU)/mL.

### Analysis of intestinal morphology

As previously described [26], the segments of the jejunum and ileum tissues were fixed in 4% formaldehyde and embedded in paraffin. Sections of 5 μm thickness were stained with hematoxylin and eosin (H&E). Photomicrographs were acquired using an Olympus IX83P2ZF microscope (Olympus, Tokyo, Japan). The morphometric analysis was performed on 10 randomly-selected, well-oriented villi and crypts per piglet. A computerized microscope-based image analyzer (Olympus dotslide Virtual Slide System, Tokyo, Japan) was used to determine the height of villus (from the tip of the villus to the villus-crypt junction) and crypt depth (from the crypt-villus junction to the base of the crypt).

### Scanning electron microscopy (SEM)

The intestinal samples of jejunum and ileum for SEM were prepared as described previously [27]. Tissues were first fixed with 2.5% glutaraldehyde overnight, washed 3 times (15 min/time) in phosphate buffer (0.1 mol/L, pH 7.0), then postfixed with 1% osmium tetroxide for 1.5 h and rinsed 3 × 15 min in PBS. Thereafter, samples were dehydrated in a graded series of ethanol (30%, 50%, 70%, 80%, 90%, 96% and 100%) for 20 min at each step. Then, the tissues were transferred to the mixture solution of alcohol and iso-amyl acetate (v:v = 1:1) for 30 min, and kept at pure iso-amyl acetate for 2 h. Then, samples were dehydrated by Hitachi Model HCP-2 critical point dryer with liquid CO_2_. In the end, dehydrated samples were coated with gold–palladium in Hitachi Model E1010 ion sputter, then observed by Hitachi Model SU-8010 scanning electron microscope (Hitachi, Tokyo, Japan).

### RNA isolation, cDNA synthesis and qRT PCR

RNA isolation, cDNA synthesis and qRT PCR of the tissues were processed according to our previous study [26]. Total RNA was extracted from the liquid nitrogen-pulverized mucosal samples with the RNAiso Plus reagent (Takara, Dalian, China) according to the manufacture’s instruction. Then, the NanoDrop ND-1000 spectrophotometer (NanoDrop Technologies, Wilmington, DE, USA) was used to detect the concentration and purity of extracted total RNA. After the dilution of all RNA samples, a total 500 ng of RNA was used to synthesis the first strand (cDNA) using PrimerScript RT reagent kit (Takara, Dalian, China).

The relative mRNA expression of tight junction (zonula occludens (ZO)-1, ZO-2, occludin (OCLN), claudin (CLDN)-2, CLDN-3, CLDN-4 and CLDN-5) in the jejunal and ileal mucosa was quantified using real-time quantitative PCR (qRT-PCR) with SYBR Premix Ex Taq kit (Takara, Dalian, China). The real time PCR reaction was performed on a CFX Opus 96 Real-time PCR instrument (Bio-Rad Laboratories, Hercules, CA, USA) after preparing a 20-μL mixture as follows: 10 μL of SYBR Premix Ex Taq (2 ×) (Takara, Dalian, China); 1 μL of cDNA, 1 μL of forward primer (10 μmol/L) and 1 μL of reverse primer (10 μmol/L), and 7 μL of water. Primers used in this study were listed in Additional file 1: Table S2. The real time PCR protocol was programmed as follows: 5 min at 95 °C; 40 cycles of 95 °C for 5 s and then 60 °C for 30 s, and generation of the dissolution curve of the gene at 95 °C for 15 s, then 60 °C for 30 s, and then 95 °C for 15 s. All samples were run in triplicate, and the relative quantification of target mRNAs was calculated using the 2^−ΔΔCt^ method using β-actin gene for normalization.

### Western-blot analysis

As previously described [26], intestinal mucosal samples were lysed and extracted by using RIPA buffer (Roche, Shanghai, China). The concentrations of protein in samples were measured by bicinchoninic acid (BCA) assay kit (Pierce, Rockford, IL, USA). The total protein samples were separated through a 10% SDS polyacrylamide gel and then transferred to a nitrocellulose membrane (Bio-Rad Laboratories, Inc., Hercules, CA, USA). The membrane was then blocked for 2 h with 5% skim milk in tris-buffered saline (TBS) at room temperature, and was subsequently incubated in primary antibodies diluted in TBS at 4 °C for overnight. Primary antibodies to rabbit polyclonal anti-ZO-2 (1:1,000 dilution), mouse monoclonal anti-OCLN (1:1,000 dilution), rabbit polyclonal anti-CLDN-3 (1:1,000 dilution), rabbit polyclonal anti-BAX (1:2,000 dilution), rabbit polyclonal anti-capase-3 (1:500 dilution), and rabbit anti-β-actin (1:10,000) from Proteintech by Thermo Fisher Scientific were used, respectively. Following 3 × 10 min washing with TBST, the membrane was incubated with horseradish peroxidase (HRP) -conjugated anti-rabbit antibody (1:10,000 dilution, Tannon, Shanghai, China) or 1:10,000 diluted HRP-conjugated anti-mouse antibody (Proteintech, Thermo Fisher Scientific, Rockford, IL, USA) for 1 h at room temperature. Tanon™ High-sig ECL Western Blotting Substrate (Tanon, Shanghai, China) was applied to the membrane for 5 min after secondary antibody incubation. The chemiluminescent signals were visualized by the Versa Doc™ imaging system. Signal intensity was quantified using Quantity One software (Bio-Rad, Hercules, CA, USA). Protein expression levels were normalized with β-actin expression level.

### Statistical analysis

All data were presented as the means ± standard deviation (SD). The data were evaluated using one-way ANOVA procedures of SPSS version 26.0 (IBM Corp., Armonk, NY, USA). Differences among groups were compared using Duncan’s multiple range test. The difference was considered significant at *P* < 0.05. Graphing was performed using GraphPad Prism version 8 (GraphPad Software, Inc., San Diego, CA, USA).

## Results

### Growth performance

**Fig. 1:**
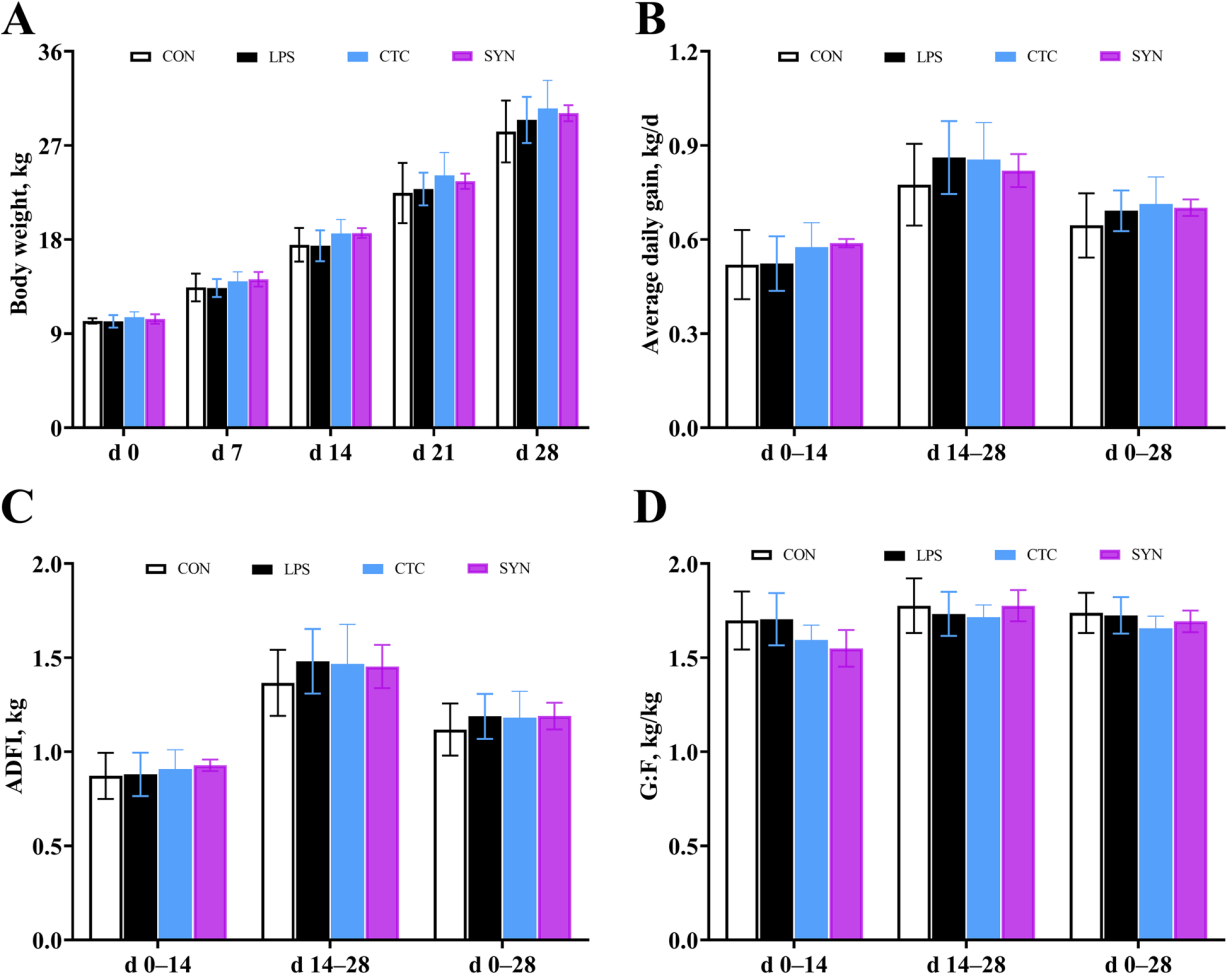
Effects of synbiotic mixture of lactulose and *Bacillus coagulans* on growth performance in weaned piglets

No significant difference (*P* > 0.05) was found on body weight (Fig. 1A), average daily gain (Fig. 1B), average daily feed intake (Fig. 1C) and feed efficacy (Fig. 1D) from different time points or periods. No difference was found on the diarrhea rate during d 0–14 (*P* > 0.05). However, diarrhea rate during the periods of d 14–28 and d 0–28 were found significantly different among the four experimental groups (Fig. S2) (*P* < 0.05). During the period of d 14–28, the CTC and SYN groups had a lower diarrhea rate than that in the CON and LPS groups (*P* < 0.05), while no difference was found between the CTC and SYN groups (*P* > 0.05). In addition, during the period of whole feeding trail (d 0–28), the CTC group had a lower diarrhea rate than that in CON and LPS groups (*P* < 0.05), and had no difference compared with the SYN group(*P* > 0.05).

### Serum levels of biomarkers for intestinal integrity

**Fig. 2:**
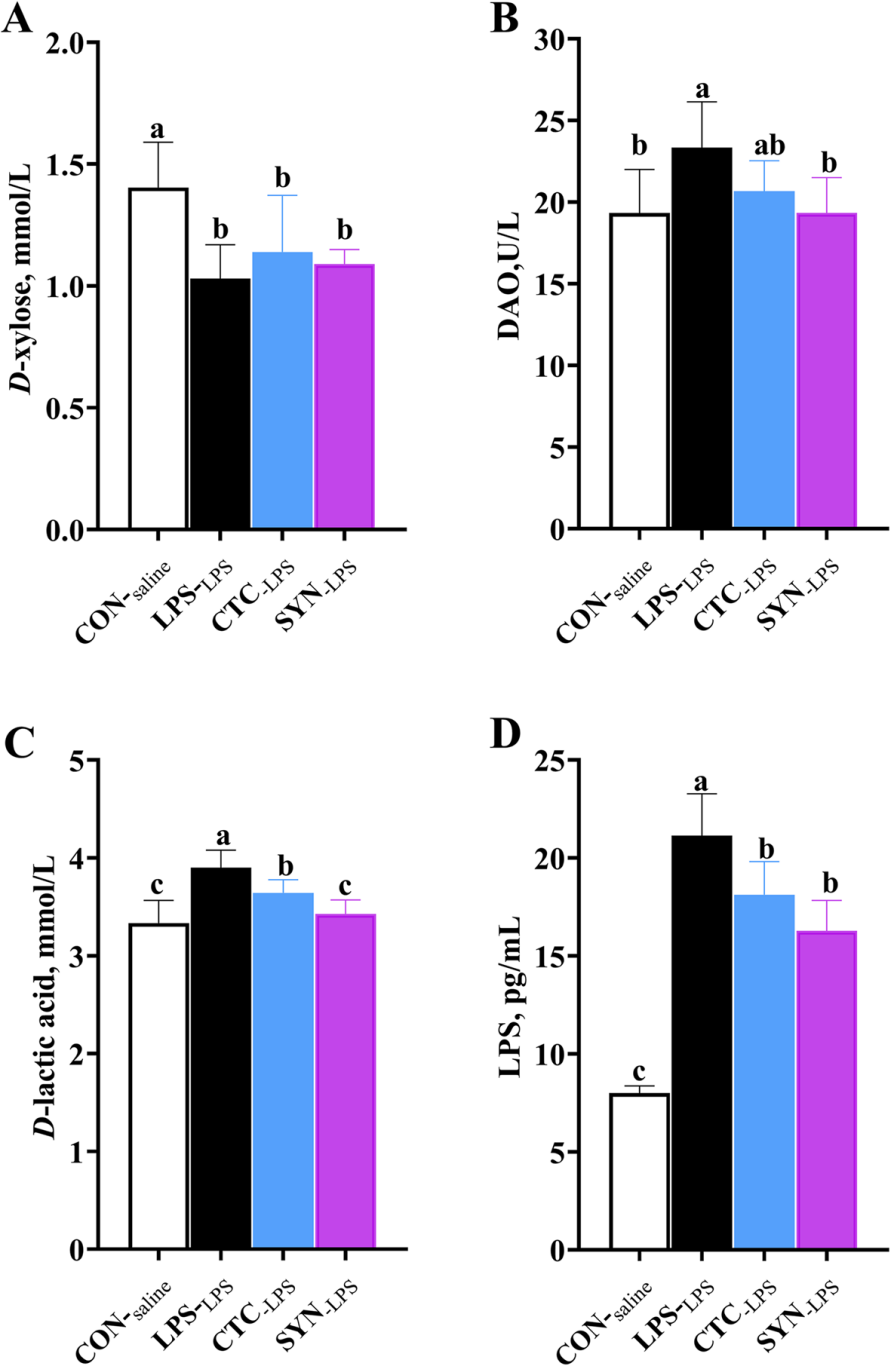
Effects of synbiotic mixture of lactulose and *Bacillus coagulans* on serum *D*-xylose*,* diamine oxidase (DAO) activities, *D*-lactic acid levels and endotoxin concentrations in weaned piglets challenged with lipopolysaccharide. CON_-saline_ (negative control), basal diet; LPS_-LPS_ (positive control), basal diet; CTC_-LPS_, basal diet + CTC (75 mg/kg); and SYN_-LPS_, basal diet + synbiotic mixture of lactulose and* Bacillus coagulans*

Four hours after saline or LPS injection, the serum biomarkers for intestinal integrity were measured, and the results are shown in Fig. 2. Compared with the CON_-saline_ group, the levels of *D*-xylose in the LPS_-LPS_ group were significantly reduced (*P* < 0.05) (Fig. 2A). In addition, the status of *D*-lactic, DAO and LPS were also obviously increased in response to LPS (*P* < 0.05) (Fig. 2B, C and D). The levels of serum *D*-xylose were found no difference (*P* > 0.05) among the three groups that were challenged with LPS. Interestingly, both the *D*-lactic acid and LPS were attenuated in either CTC_-LPS_ or SYN_-LPS_ groups(*P* < 0.05) (Fig. 2C and D). Moreover, the SYN_-LPS_ group also had lower activity of serum DAO compared with the LPS_-LPS_ group (*P* < 0.05).

### Intestinal morphological response

**Fig. 3:**
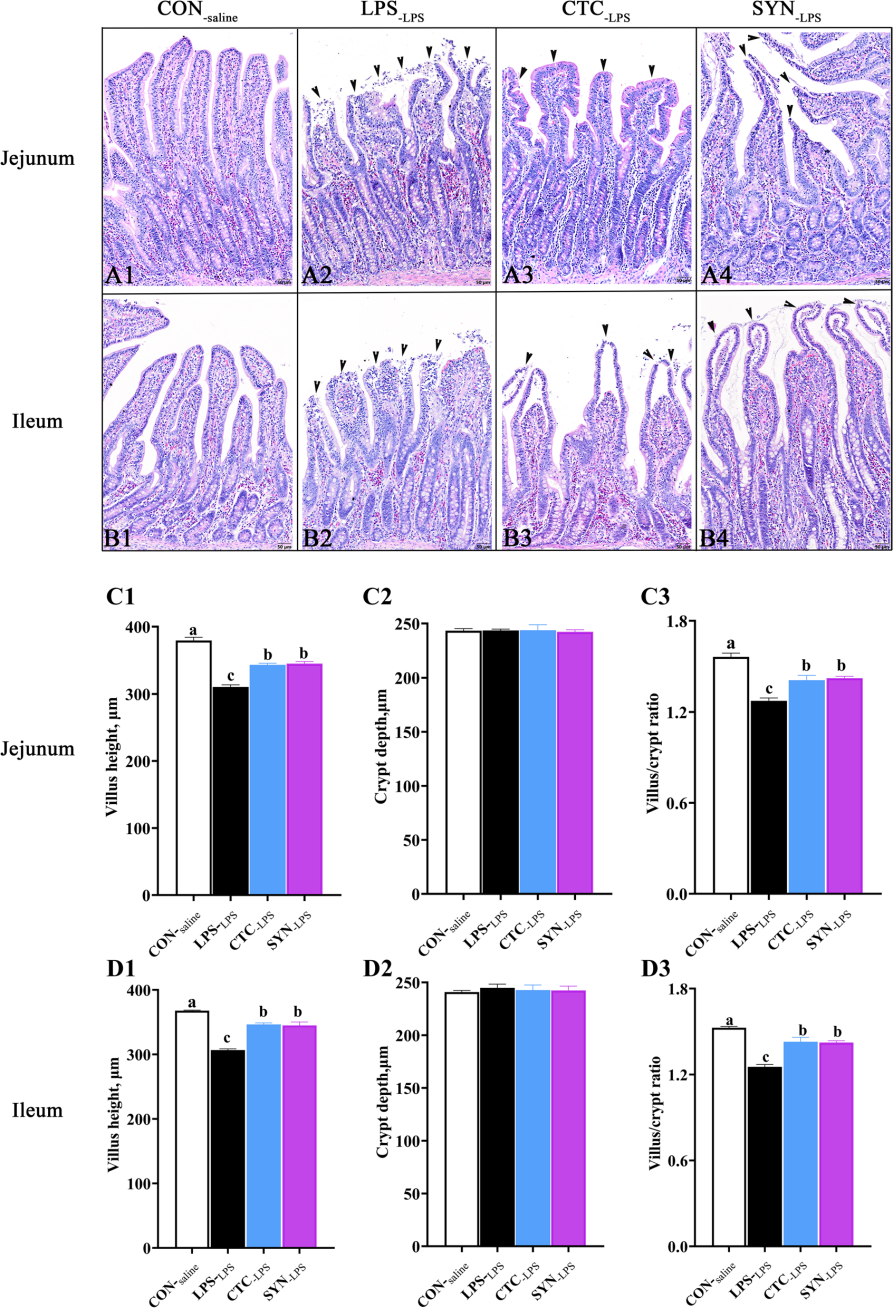
Effects of synbiotic mixture of lactulose and *Bacillus coagulans* on histological changes and morphology of jejunum and ileum in piglets challenged with lipopolysaccharide. Representative haematoxylin & eosin (H&E) staining images were obtained at 200 × magnification with black bar = 50 μm. **A**1–4 Jejunum tissue images; **B**1–4 Ileum tissue images; **C**1–3 Jejunum morphology; **D**1–3 Ileum morphology. CON_-saline_ (negative control), basal diet; LPS_-LPS_ (positive control), basal diet; CTC_-LPS_, basal diet + CTC (75 mg/kg); and SYN_-LPS_, basal diet + synbiotic mixture of lactulose and* Bacillus coagulans*

As black arrows indicated in Fig. 3, LPS challenge significantly caused shedding and swelling the villus tip of jejunum and ileum in weaned piglets (Fig. 3A2 and B2), with many truncated epithelial cells in the tip of the villus. In contrast, piglets either administrated CTC or synbiotic mixture of lactulose and *Bacillus coagulans* improved the structure of the intestinal villi to a large extent in the jejunum (Fig. 3A3 and A4) and ileum (Fig. 3B3 and B4). The villus height, crypt depth and ratio of villus/crypt were also measured (Fig. 3C and D). LPS challenge significantly decreased the villus height and villus/crypt ratio in both jejunum (Fig. 3C1 and D1) and ileum (Fig. 3C3 and D3) (*P* < 0.05), while crypt depth was not influenced by LPS injection (Fig. 3C2 and D2). Compared with the LPS_-LPS_ group, both CTC_-LPS_ and SYN_-LPS_ groups had higher villus height and ratio of villus/crypt depth in jejunum and ileum, while no difference was found between the CTC_-LPS_ and SYN_-LPS_ groups (*P* < 0.05).

**Fig. 4:**
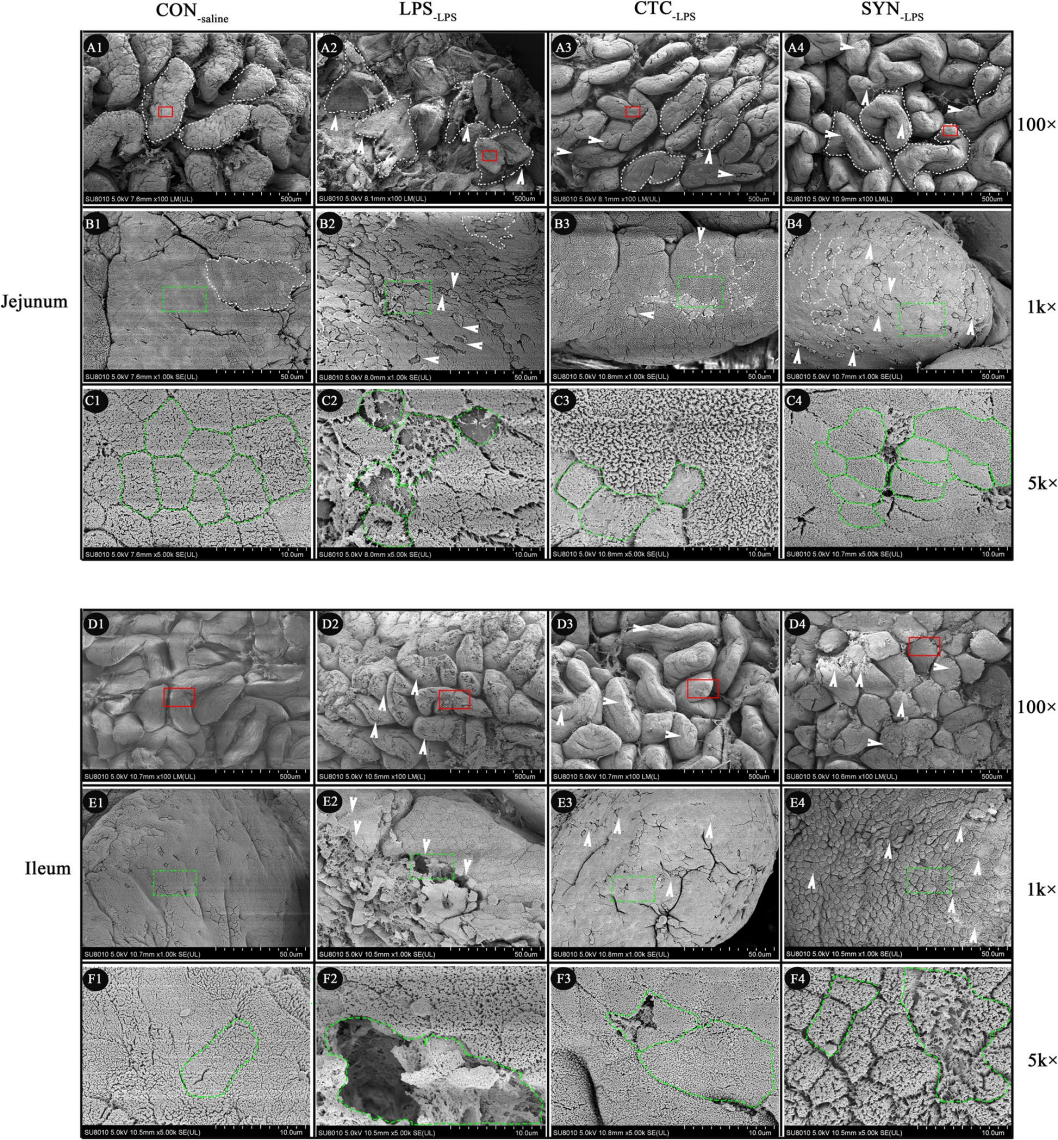
Scanning electron microscopy (SEM) images of jejunal and ileal mucosa in weaned piglets. Normal jejunal (**A1**, **B1** and **C1**) and ileal (**D1**, **E1** and **F1**) mucosa with cilia carpet and arranged microvilli. Brush border collapsed (white arrow), non-ciliated cells (green circle), cell loss (green circle) and many microvilli disappeared (green circle) were obvious found in the tissues of both jejunum and ileum among the piglets challenged with LPS. CON_-saline_ (negative control), basal diet; LPS_-LPS_ (positive control), basal diet; CTC_-LPS_, basal diet + CTC (75 mg/kg); and SYN_-LPS_, basal diet + synbiotic mixture of lactulose and* Bacillus coagulans*

SEM analysis was used to further observe the ultrastructure of the small intestine (jejunum and ileum) among the four experimental groups. The SEM results were basically consisted with the data from serum biomarkers and morphological analysis. As indicated by arrows (white color) and outlines (green color) in Fig. 4, the intestinal villus and microvilli structure of jejunum (Fig. 4A1, B1 and C1) and ileum (Fig. 4D1, E1 and F1) was strictly ordered with a smooth surface and clear structure under the CON_-saline_ group. Notably, LPS injection causing the strictly ordered brush border collapsed and many microvilli separated in the tissues of both jejunum (Fig. 4A2, B2 and C2) and ileum (Fig. 4D2, E2 and F2). For the jejunum and ileum tissue under the CTC_-LPS_ and SYN_-LPS_ groups, although visible damage was also found in both jejunum (Fig. 4A3, B3, C3, A4, B4 and C4) and ileum (Fig. 4D3, E3, F3, D4, E4 and F4), the basic intestinal microvillus structure appeared to have significantly alleviated compared with the LPS_-LPS_ group.

### mRNA and protein changes related to barrier function

**Fig. 5:**
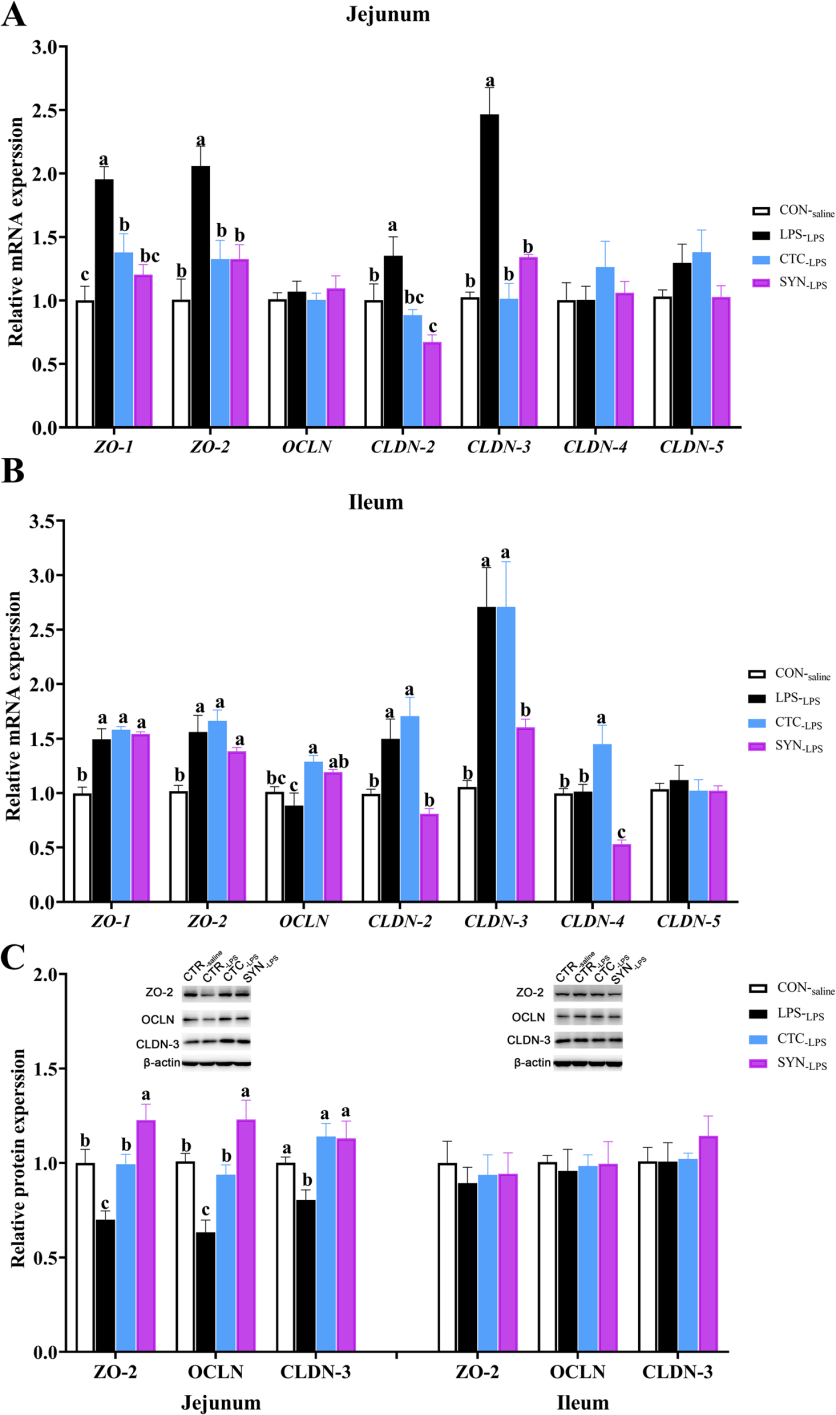
Effects of synbiotic mixture of lactulose and *Bacillus* *coagulans* on relative mRNA gene (**A** and **B**) and protein (**C**) expression levels related to tight junction in jejunum (**A**), and ileum (**C**) of weaned piglets challenged with LPS. Each column represents the mean values (*n* = 6), with their standard error of mean (SEM) represented by vertical bars. Letters above the bars not sharing the same lower (*P* < 0.05) or upper (*P* < 0.01) case superscript are significantly different. ZO-1 = zonula occluden 1, ZO-2 = zonula cooluden 2, OCLN = occludin, CLDN-2 = claudin 2, CLDN-3 = claudin 3, CLDN-4 = claudin 4, CLDN-5 = claudin 5. CON_-saline_ (negative control), basal diet; LPS_-LPS_ (positive control), basal diet; CTC_-LPS_, basal diet + CTC (75 mg/kg); and SYN_-LPS_, basal diet + synbiotic mixture of lactulose and *Bacillus coagulans*

To verify the remission of intestinal structure caused by LPS was related to the expression of tight junction, the relative expression levels of genes and proteins for representative tight junction in the jejunum and ileum were analyzed. Compared with the CON_-saline_ group, mRNA relative expression levels of *OCLN*, *CLDN-4* and *CLDN-5* in both jejunum and ileum were not influenced by LPS challenge or dietary treatment (*P* > 0.05) (Fig. 5A and B). However, mRNA levels of *ZO-1*, ZO-2, *CLDN-2* and *CLDN-3* in both jejunum and ileum were significantly increased responding to the LPS injection (*P* < 0.05) (Fig. 5A and B). Dietary supplementation of either CTC or synbiotic mixture of lactulose and *Bacillus coagulans* was found lower the mRNA levels of *ZO-1*, ZO-2, *CLDN-2* and *CLDN-3* in jejunum compared with the LPS group (*P* < 0.05) (Fig. 5A). In the ileum, mRNA levels of *ZO-1*, ZO-2, and *CLDN-5* had no difference among the LPS_-LPS_, CTC_-LPS_ and SYN_-LPS_ groups (*P* > 0.05) (Fig. 5B). However, mRNA levels of *CLDN-2* and *CLDN-3* in the SYN_-LPS_ group were lower than that in the LPS_-LPS_ group (*P* < 0.05), while CTC had no influenced in the relative expression of these genes. For the mRNA levels of ileum *OCLN*, both CTC_-LPS_ and SYN_-LPS_ groups were found higher than that in the LPS_-LPS_ group (*P* < 0.05), while no difference was found between the CTC_-LPS_ and SYN_-LPS_ groups (*P* > 0.05). For the ileum *CLDN-4* gene expression, the CTC_-LPS_ group was higher than that in both LPS_-LPS_ and SYN_-LPS_ groups (*P* < 0.05), while the SYN_-LPS_ group was lower than that in the LPS_-LPS_ group (*P* < 0.05).

Based on the results of mRNA expression in jejunum and ileum, relative protein expression levels of ZO-2, OCLN and CLDN-3 were also chosen to exam the status of epithelial barrier function among the four experimental groups (Fig. 5C). In the ileum, no difference was found in these relative protein expression between the CON_-saline_ and LPS_-LPS_ groups (*P* > 0.05), or among the LPS_-LPS_, CTC_-LPS_ and SYN_-LPS_ groups (*P* > 0.05) (Fig. 5C). In the jejunum, the relative protein levels of ZO-2, OCLN and CLDN-3 in the LPS_-LPS_ group were significantly lower than that in the CON_-saline_ group (*P* < 0.05) (Fig. 5C). Both CTC and SYN treatments were found significantly increased the jejunal ZO-2, OCLN and CLDN-3 protein expression than that in the LPS_-LPS_ group (*P* < 0.05) (Fig. 5C). Moreover, relative protein levels of jejunal ZO-2 and OCLN in the SYN_-LPS_ group were also higher than that in the CTC_-LPS_ group (*P* < 0.05). However, no difference was found on the jejunal CLDN-3 protein levels between the CTC_-LPS_ and SYN_-LPS_ groups (*P* > 0.05).

### Intestinal apoptosis status detected by TUNEL and Western Blot

**Fig. 6:**
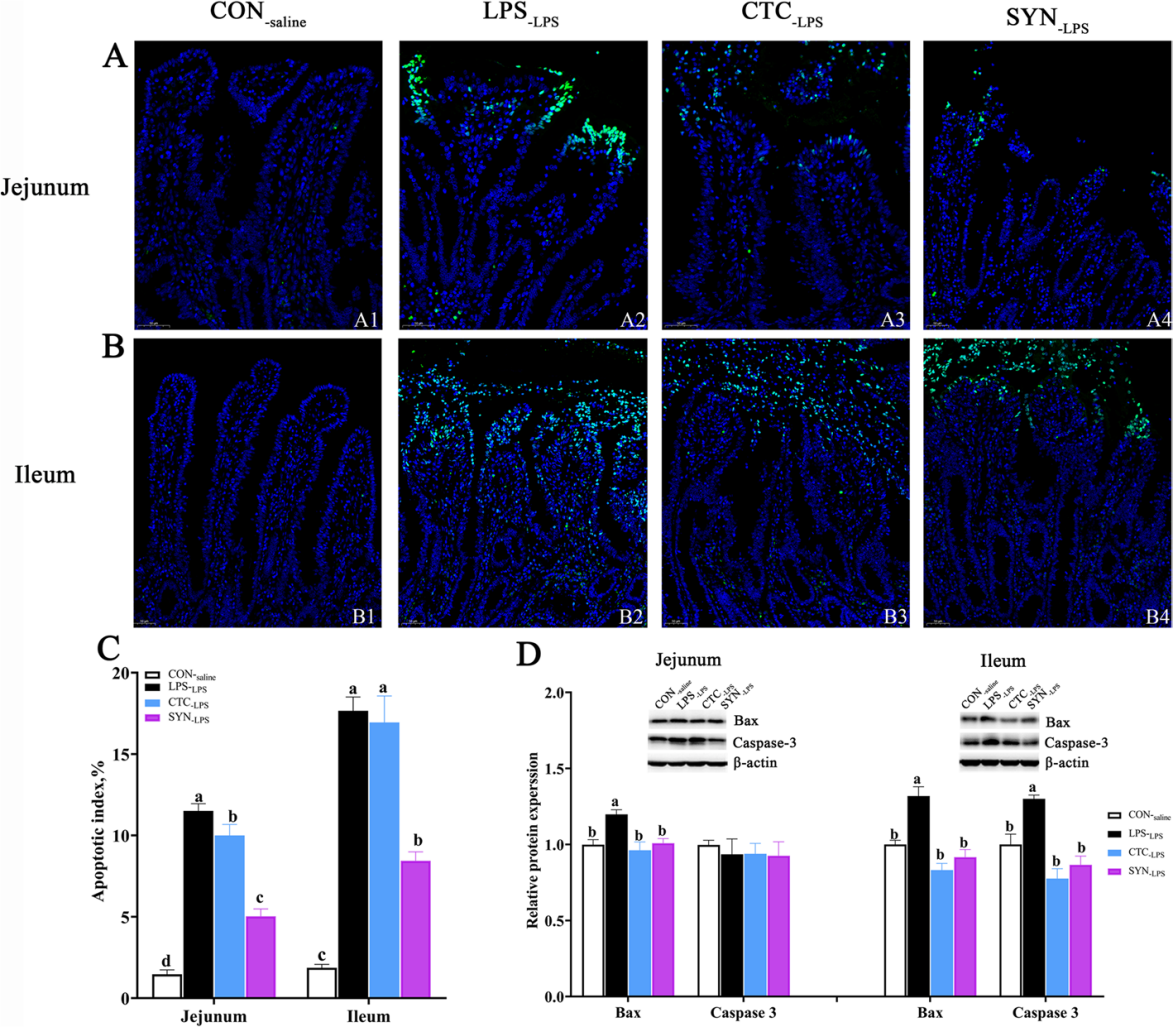
Effects of synbiotic mixture of lactulose and *Bacillus coagulans* on the percentage of apoptosis intestinal epithelial cells by the TUNEL assay, and relative levels of apoptosis-related protein in intestinal mucosa in weaned piglets challenged with LPS. Representative TUNEL stained paraffin sections from jejunum (**A**1–4) and ileum (**B**1–4) tissue. **B** Effects of synbiotic mixture of lactulose and *Bacillus coagulans* on the percentage of jejunal and ileal apoptosis ratio in piglets challenged with LPS. **C** Effects of synbiotic mixture of lactulose and *Bacillus coagulans* on relative protein expression levels related to apoptosis in jejunum and ileum of piglets challenged with LPS. Each column represents the mean values (*n* = 6), with their standard error of mean (SEM) represented by vertical bars. Letters above the bars not sharing the same lower (*P* < 0.05) or upper (*P* < 0.01) case superscript are significantly different. CON_-saline_ (negative control), basal diet; LPS_-LPS_ (positive control), basal diet; CTC_-LPS_, basal diet + CTC (75 mg/kg); and SYN_-LPS_, basal diet + synbiotic mixture of lactulose and *Bacillus coagulans*

Representative observations of TUNEL staining in the jejunum and ileum from the four experimental groups were shown in Fig. 6. Following in situ labeling, stained epithelial cells from the jejunum and ileum were undergoing aggressive apoptosis in piglets challenged with LPS under microscopic examination (Fig. 6A2 and B2). A number of TUNEL-positive cells were also found in piglets from CTC_-LPS_ and SYN_-LPS_ groups, respectively. The apoptosis index for the quantification of TUNEL-positive cells is shown in Fig. 6C. Compared with the CON_-saline_ group, piglets challenged with LPS were found to have a higher apoptosis index both in jejunum and ileum (*P* < 0.05). Compared with the LPS_-LPS_ group, dietary treatment with either CTC or SYN treatment lowered the apoptosis index both in jejunum and ileum (*P* < 0.05). In addition, the SYN_-LPS_ group also had a lower apoptosis index than that in the CTC_-LPS_ group.

The relative expression of apoptosis-related proteins Bax and caspase-3 in both jejunum and ileum were also measured to further confirm the remission effects of SYN on the intestinal apoptosis. Compared with the CON_-saline_ group, LPS challenge resulted in a significant increase in protein expression of Bax in both jejunum and ileum, and caspase-3 in the ileum (Fig. 6D). Compared with the LPS_-LPS_ group, both CTC and SYN treatments had lower protein expression of Bax either in the jejunum or ileum (Fig. 6D), while no difference was found among the CTC_-LPS_ and SYN_-LPS_ groups. In addition, the relative protein expression of caspase-3 in the CTC_-LPS_ and SYN_-LPS_ groups was also found significantly lower than that in the LPS_-LPS_ group (*P* < 0.05), while no difference was found among the CTC_-LPS_ and SYN_-LPS_ groups.

## Discussion

The current study was to designed to evaluate the effects of synbiotic mixture with lactulose and *Bacillus coagulans* supplementation as a replacement of antimicrobial growth-promoter (CTC) on growth performance and the resilience of intestine to immune stress in weaned piglets. Before LPS injection, the performance of piglets fed CTC or SYN diet did not differ from that of piglet fed the basal diet. In fact, considerable variability in response to CTC supplementation has been reported [28]. Many studies have shown no promoting growth performance effects in piglets fed with AGPs (including chlortetracycline, tiamulin, bacitracin, colistin, aureomycin, avilamycin, nosiheptide, olaquindox, oxytetracycline, lincospectin, apramycin and tylosin) diet in the absence of health problems [28]. In addition, the diarrhea incidence in the SYN group was similar to the CTC group, which significantly lower than those in the CON group during the period of d 14 to 28 (Fig. S2). These findings consisted with previous studies shown that the application of either *Bacillus coagulans* or lactulose obtained good results in animal husbandry [11]. For example, feeding 600 g *Bacillus coagulans* (8.0 × 10^9^ CFU/g, spore rate > 98%) to weaned piglets for 28 d had positive effects on growth performance (higher ADG and lower ratio of F:G) [13]. Moreover, dietary supplementation with 0.1% lactulose improved the growth performance (greater ADG and G/F) in weaning piglets [15]. Although no promotion effects were found on the growth performance of SYN, the diarrhea rate was significantly attenuated by SYN. This may attribute to the production of secondly metabolites of microbiota such as exogenous enzymes, vitamins, amino acids, short-chain fatty acids and some unknown factors through the microbiota by *Bacillus coagulans* [29] and lactulose supplementation [16, 30].

The intestinal epithelial barrier acts as a selectively permeable barrier against the exogenous substances such as toxins, antigens and enteric microflora [31]. The integrity of intestinal barrier can be indirectly reflected by serum indicators such as DAO activity, absorption of *D*-xylose, status of *D*-lactic acid and LPS. Previous studies have shown that intraperitoneal injection of LPS (100 μg/kg BW) in weaned piglets would disrupt the intestinal epithelial barrier resulting increasement of the permeability of gut [32]. As expected, the activities DAO, levels of *D*-lactic acid and LPS in the serum of the LPS_-LPS_ group were significantly higher than those in the CON_-saline_ group. Moreover, the *D*-xylose concentration was found significantly lower by LPS challenge. These findings were coincident with previous well-document studies [23, 24] and indicated an acute stress model built successfully in our study. Interestingly, both CTC and synbiotic mixture of lactulose and *Bacillus coagulans* supplementation normalized the changes of serum intestinal barrier biomarkers under LPS challenge. A previous study has shown that the addition of *Bacillus coagulans* (2 × 10^6^ CFU/g and 2 × 10^7^ CFU/g) to piglets’ feed for 21 d could effectively reduce plasma DAO activity [33]. Supplementation of lactulose (500 mg/kg) was also found significantly reduced serum DAO activity, LPS concentration and *D*-lactate levels in weaned piglets fed *Fusarium* mycotoxin-contaminated diet [26]. *D*-xylose is a poorly metabolized pentose, which its absorption test has been proven to be a reliable indicator for intestinal function [34]. In the present study, no significant difference in serum *D*-xylose levels was found among the three groups challenged with LPS. In addition, piglets in the CON_-saline_ group had higher serum *D*-xylose levels than that in the three LPS challenged groups. This may cause by LPS injection induced strong stress reactions, such as vomiting, acute diarrhea, etc., resulting the *D*-xylose solution excreted with the vomit.

The health of the intestinal tract is the physiological basis to meet the growth potential of piglets. Villus height, crypt depth, and villus height:crypt depth ratio is strongly related to functions of digestion, absorption and cell maturation [35]. LPS stimulation can cause intestinal morphological changes and barrier function impairments in weaned piglets, resulting in increased intestinal permeability [32]. Chen et al. [36] showed that intraperitoneal injection of LPS (100 μg/kg BW) for 3 h in weaned piglets significantly reduced the villus height and villus-to-crypto ratio of the small intestine. In addition, it has also been reported that LPS stimulation (100 μg/kg BW) for 24 h resulted in a significant decrease in jejunal villus height and villus height:crypt depth ratio and a significant increase in jejunal and ileal crypt depth in weaned piglets [37]. In this experiment, it was also found that the villus height and villus height:crypt depth ratio of the small intestine of weaned piglets were significantly decreased after intraperitoneal injection of LPS for 4 h. Scanning electron microscope observation of intestinal epithelium also showed that compared with the CON_-saline_ group, the small intestinal villi of the jejunum and ileum tissue in the CON_-LPS_ group were severely damaged, mainly manifested as atrophy, damage and rupture of the intestinal villi, and the microvilli were tufted, falling off, not neatly arranged. The abnormality of intestinal tissue morphology and barrier function induced by LPS is basically consistent with the findings of Fan et al. [25]. Studies have shown that dietary supplementation of *Bacillus coagulans* (2 × 10^6^ CFU/g and 2 × 10^7^ CFU/g) to weaned piglets (21 days old) for 21 d could effectively reduce diarrhea, while significantly improving intestinal morphology, including jejunal crypts and depth decreased, the height of the ileal villi increased, and the ratio of empty to ileal villi also increased [33]. Guerra-Ordaz et al. [38] found that continuous 17 d supplementation of 10 g/kg lactulose enhanced ETEC K88 stimulation in weaned piglets by selectively increasing the number and/or activity of beneficial bacteria, improving ileal villus height and goblet cell number intestinal mucosal defenses. Previous studies have also shown that chlortetracycline can significantly increase the intestinal villus height and villus height: crypt depth ratio in weaned piglets, thereby alleviating intestinal morphological damage caused by ETEC or LPS stimulation [39]. In this experiment, the observation results of intestinal tissue morphology all showed the protective effect of CTC and synbiotic mixture of lactulose and *Bacillus coagulans* on intestinal morphology of weaned piglets, including significantly inhibiting LPS-induced small intestinal villus height, villus height:crypt depth ratio decreased, reduced intestinal villi damage and microvillus shedding. It can be speculated that supplementation of synbiotic containing lactulose and *Bacillus coagulans* might attribute to the modulation of gut microbiota and its metabolites. However, the underlying mechanism of the synbiotic mixture of *Bacillus coagulans* and lactulose improving the resistance of the intestine against LPS challenge remains to be investigated.

Numerous studies have shown that LPS can disrupt the intestinal barrier integrity and reduce intestinal tight junction mRNA expression and protein levels [5, 40]. Wang et al. found that the expression level of *CLDN-1* mRNA in the jejunal mucosa was significantly down-regulated 4 h after intraperitoneal injection of 100 μg/kg BW LPS in weaned piglets, while the mRNA expression level of *OCLN* was not significantly affected [41]. Xiao et al. [42] also found that 4 h after LPS stimulation (100 μg/kg BW) could induce a significant decrease in the protein levels of ZO-1, OCLN and CLDN-1 in the jejunal mucosa of weaned piglets, which was consistent with the jejunal tight junction protein level in this experiment. Moreover, we also found that CTC shown protective effects on the intestinal injury. As a matter of fact, Hu et al. [43] showed that chlortetracycline (50 mg/kg) could significantly inhibit the LPS-induced reduction of OCLN and ZO-1 gene expression levels and protein expression in the ileal mucosa of weaned piglets. In this experiment, dietary synbiotic mixture of lactulose and *Bacillus coagulans* not only significantly alleviated the increasement of intestinal permeability caused by LPS, but also effectively alleviated the up-regulation of mRNA expression levels and down-regulation of protein levels of intestinal tight junction induced by LPS. It also needs to mention that there is a different response of protein expression in jejunum and ileum, which is contrary to the data previously published [44]. However, no clear explanation for the contradicting results between the two segments of intestinal. It can be speculated that this could be related to many factors such as immune challenge dose, sampling time, the age of piglets. Previous studies showed that lactulose can not only selectively stimulate the growth of beneficial flora (mainly including *Lactobacillus* and *Bifidobacterium*), but also significantly reduce the number of *Salmonella*, *Escherichia*, and *Clostridium* in the gastrointestinal tract and the production of toxic metabolites [45, 46]; In addition, it can also accelerate intestinal peristalsis and promote the excretion of toxins from the enteric cavity [47]. Probiotics can prevent pathogen- or chemical-induced intestinal barrier dysfunction by stimulating mucin and secretory immunoglobulin A secretion and enhancing intestinal tight junction protein levels [48]. Wang et al. [49] administered *Bacillus coagulans* (1 × 10^9^ CFU) to SPF mice with dextran sulfate sodium-induced colitis and found that *Bacillus coagulans* could significantly upregulate the expression levels of colonic *OCLN* and mucin-2 mRNA, thereby enhancing the intestinal mucus layer and resists the invasion of microorganisms. Therefore, the synbiotic mixture of lactulose and *Bacillus coagulans* may protect the integrity of the intestinal barrier and improve its resistance to LPS challenge by improving the balance of intestinal flora in piglets, maintaining homeostasis, and improving the level of tight junction proteins.

Epithelial turnover, including cell proliferation and apoptosis, play a critical role in the maintenance of intestinal integrity and function. Cell apoptosis has been defined as a form of programmed cell death controlled by various genes to maintain the homeostasis of body tissue. Uncontrolled or aggressive apoptosis contributes to dysfunction of the intestine and is commonly observed in various intestinal diseases. It was well known that the activation of caspases-3 and Bax was the feature markers for the mitochondria-dependent apoptosis pathway. In the present study, both CTC and SYN supplementation reduced apoptosis, ameliorated the regulation of caspase-3 (only in jejunum) and Bax in intestinal induced by LPS challenge. In support of our result, previous reports demonstrated that the oral administration of 500 mg/kg BW of lactulose (twice daily) significantly down-regulated the apoptosis index, apoptosis-related gene expression in the jejunum (Bcl-2 and caspase-3) and ileum (caspase-3) in piglets which fed Fusarium mycotoxins contaminated diet [26]. In a methamphetamine (METH) -induced neurotoxicity model, lactulose (5.3 g/kg, oral gavage, 12-h interval) administration for 2 d prior to the METH administration was found attenuated the METH-induced neurotoxicity by alleviating the impaired autophagy, stabilizing the perturbed antioxidant system and suppressing apoptosis in rat striatum [50]. Interestingly, *Bacillus coagulans* was also found could maintain mouse intestine model cells in a normal proliferation ratio and reduce the reactive oxygen species and apoptosis ratios under lead exposure conditions [51]. In addition, the protein level of caspase 3 in the jejunum was not influenced by LPS injection or dietary treatment, regardless of apoptosis index was different among the groups. We do not have a clear explanation for this anomaly, and further studies may be needed to further explore the underlying mechanism. In general, these results suggested that supplementation of synbiotic containing lactulose with *Bacillus coagulans* may protect intestinal integrity by inhibiting the apoptosis of epithelial cell. And the underlying mechanism of the synbiotic mixture of lactulose and *Bacillus coagulans* regulation of cell apoptosis needs further assessment. Due to the replication limit, further experiments involved a large number of replications are definitely needed to be explored.

## Conclusion

In conclusion, our findings suggest that dietary synbiotic containing lactulose and *Bacillus coagulans* resulted in a lower diarrhea rate, greater resilience to LPS-induced intestinal morphology damage, barrier dysfunction and apoptosis in piglets. Those results indicate that the synbiotic mixture of lactulose and *Bacillus coagulans* as a novel additive shown beneficial effects on the performance and resilience to acute immune stress in weaned piglets.

## Supplementary Information

**Additional file 1: Fig. S1.** Flow chart of this study. **Fig. S2.** Effects of synbiotic mixture of lactulose and Bacillus coagulans on the diarrhea in piglets during the feeding trial. **Table S1.** Ingredient composition and nutrient contents of the basal experimental diet. **Table S2.** List of primers used in this study.

## Data Availability

The datasets used and/or analyzed during the current study are available from the corresponding author on reasonable request.

## Abbreviations

AGPs: Antimicrobial growth promoters
BAC: Bicinchoninic acid
CLDN-2: Claudin 2
CLDN-3: Claudin 3
CLDN-4: Claudin 4
CLDN-5: Claudin 5
CTC: Chlortetracycline
DAI: Diamine oxidase
GI: Gastrointestinal
H&E: Hematoxylin and eosin
JAMs: Junctgional adhesion molecules
LPS: Lipopolysaccharide
OCLN: Occludin
TJs: Tight junctions
TUNEL: Terminal deoxynucleotidyl transferase dUTP nick end labeling
ZOs: Zonula occludins
ZO-1: Zonula occluden 1
ZO-2: Zonula occluden 2

## References

[1] Wells JM, Gao Y, de Groot N, Vonk MM, Ulfman L, van Neerven RJJ (2022). Babies, bugs, and barriers: dietary modulation of intestinal barrier function in early life. Annu Rev Nutr.

[2] Rescigno M (2011). The intestinal epithelial barrier in the control of homeostasis and immunity. Trends Immunol.

[3] Kirschner N, Houdek P, Fromm M, Moll I, Brandner JM (2010). Tight junctions form a barrier in human epidermis. Eur J Cell Biol.

[4] Ma YD, Lv QF, Zhao DD, Wang JJ, Fu Y, Li C (2022). Intervention effect of taurine on LPS-Induced intestinal mechanical barrier injury in piglets. Adv Exp Med Biol.

[5] Guo J, Liang T, Chen H, Li X, Ren X, Wang X (2022). Glutamate attenuates lipopolysaccharide induced intestinal barrier injury by regulating corticotropin-releasing factor pathway in weaned pigs. Anim Biosci.

[6] Campbell JM, Crenshaw JD, Polo J (2013). The biological stress of early weaned piglets. J Anim Sci Biotechnol.

[7] Rhouma M, Fairbrother JM, Beaudry F, Letellier A (2017). Post weaning diarrhea in pigs: risk factors and non-colistin-based control strategies. Acta Vet Scand.

[8] Li J (2017). Current status and prospects for in-feed antibiotics in the different stages of pork production - A review. Asian-Australas J Anim Sci.

[9] Heo JM, Opapeju FO, Pluske JR, Kim JC, Hampson DJ, Nyachoti CM (2013). Gastrointestinal health and function in weaned pigs: a review of feeding strategies to control post-weaning diarrhoea without using in-feed antimicrobial compounds. J Anim Physiol Anim Nutr (Berl).

[10] Rodrigues G, Maximiano MR, Franco OL (2021). Antimicrobial peptides used as growth promoters in livestock production. Appl Microbiol Biotechnol.

[11] Zhou YH, Zeng ZH, Xu YB, Ying JF, Wang BK, Majeed M, et al. Application of Bacillus coagulans in Animal Husbandry and Its Underlying Mechanisms. Animals. 2020;10(3):9. 10.3390/ani10030454. 10.3390/ani10030454 PMC7143728 32182789

[12] Pu J, Chen D, Tian G, He J, Zheng P, Mao X (2020). Effects of benzoic acid, Bacillus coagulans and oregano oil combined supplementation on growth performance, immune status and intestinal barrier integrity of weaned piglets. Anim Nutr.

[13] Sun T, Miao H, Zhang C, Wang Y, Liu S, Jiao P (2022). Effect of dietary Bacillus coagulans on the performance and intestinal microbiota of weaned piglets. Animal.

[14] Ruszkowski J, Witkowski JM (2019). Lactulose: Patient- and dose-dependent prebiotic properties in humans. Anaerobe.

[15] Cho JH, Kim IH (2015). Effects of lactulose supplementation on growth performance, nutrient digestibility, blood profiles, faecal microbial shedding, faecal score and faecal noxious gas emission in weanling pigs. J Appl Anim Res.

[16] Zheng W, Ji X, Zhang Q, Du W, Wei Q, Yao W. Hydrogen-rich water and lactulose protect against growth suppression and oxidative stress in female piglets fed Fusarium toxins contaminated diets. Toxins (Basel). 2018;10(6):228. 10.3390/toxins10060228. 10.3390/toxins10060228 PMC6024318 29867031

[17] Liu R, Qiao Y, Huang G, Qian W, Xiong J, Wang X (2022). Effect of synbiotic containing Bacillus coagulans and lactulose on gut health in mice with DSS-induced ulcerative colitis. Acta Microbiologica Sinica.

[18] Williams JM, Duckworth CA, Watson AJ, Frey MR, Miguel JC, Burkitt MD (2013). A mouse model of pathological small intestinal epithelial cell apoptosis and shedding induced by systemic administration of lipopolysaccharide. Dis Model Mech.

[19] Liu Y, Chen F, Odle J, Lin X, Jacobi SK, Zhu H (2012). Fish oil enhances intestinal integrity and inhibits TLR4 and NOD2 signaling pathways in weaned pigs after LPS challenge. J Nutr.

[20] National Research Council. Nutrient Requirements of Swine: Eleventh Revised Edition. Washington, DC: The National Academies Press; 2012. 10.17226/13298. https://nap.nationalacademies.org/catalog/13298/nutrient-requirements-of-swine-eleventh-revised-edition.

[21] Walsh AM, Sweeney T, O'Shea CJ, Doyle DN, O'Doherty JV (2012). Effect of supplementing different ratios of laminarin and fucoidan in the diet of the weanling piglet on performance, nutrient digestibility, and fecal scoring. J Anim Sci.

[22] Zheng W, Ji X, Zhang Q, Yao W. Intestinal microbiota ecological response to oral administrations of hydrogen-rich water and lactulose in female piglets fed a *Fusarium* toxin-contaminated diet. Toxins (Basel). 2018;10(6):246. 10.3390/toxins10060246. 10.3390/toxins10060246 PMC6024725 29914163

[23] Leng W, Liu Y, Shi H, Li S, Zhu H, Pi D (2014). Aspartate alleviates liver injury and regulates mRNA expressions of TLR4 and NOD signaling-related genes in weaned pigs after lipopolysaccharide challenge. J Nutr Biochem.

[24] Zhang H, Zhang B, Zhang X, Wang X, Wu K, Guan Q (2017). Effects of cathelicidin-derived peptide from reptiles on lipopolysaccharide-induced intestinal inflammation in weaned piglets. Vet Immunol Immunopathol.

[25] Fan C, Han J, Liu X, Zhang F, Long Y, Xie Q (2019). Modulation of hypoxia-inducible factor-1alpha/cyclo-oxygenase-2 pathway associated with attenuation of intestinal mucosa inflammatory damage by Acanthopanax senticosus polysaccharides in lipopolysaccharide-challenged piglets. Br J Nutr.

[26] Ji X, Zhang Q, Zheng W, Yao W (2019). Morphological and molecular response of small intestine to lactulose and hydrogen-rich water in female piglets fed Fusarium mycotoxins contaminated diet. J Anim Sci Biotechnol.

[27] Cao S, Hou L, Sun L, Gao J, Gao K, Yang X (2022). Intestinal morphology and immune profiles are altered in piglets by early-weaning. Int Immunopharmacol.

[28] Wang H, Long W, Chadwick D, Zhang X, Zhang S, Piao X (2022). Dietary acidifiers as an alternative to antibiotics for promoting pig growth performance: A systematic review and meta-analysis. Animal Feed Sci Technol.

[29] Gu SB, Zhao LN, Wu Y, Li SC, Sun JR, Huang JF (2015). Potential probiotic attributes of a new strain of Bacillus coagulans CGMCC 9951 isolated from healthy piglet feces. World J Microbiol Biotechnol.

[30] Chen X, Zuo Q, Hai Y, Sun XJ (2011). Lactulose: an indirect antioxidant ameliorating inflammatory bowel disease by increasing hydrogen production. Med Hypotheses.

[31] Groschwitz KR, Hogan SP (2009). Intestinal barrier function: molecular regulation and disease pathogenesis. J Allergy Clin Immunol..

[32] Huang S, Zhang S, Chen L, Pan X, Wen Z, Chen Y (2022). Lipopolysaccharide induced intestinal epithelial injury: a novel organoids-based model for sepsis in vitro. Chin Med J (Engl).

[33] Wu T, Zhang Y, Lv Y, Li P, Yi D, Wang L, et al. Beneficial Impact and Molecular Mechanism of *Bacillus coagulans* on Piglets' Intestine. Int J Mol Sci. 2018;19(7):2084. 10.3390/ijms19072084 10.3390/ijms19072084 PMC6073773 30021943

[34] Doerfler RE, Cain LD, Edens FW, Parkhurst CR, Qureshi MA, Havenstein GB (2000). D-xylose absorption as a measurement of malabsorption in poult enteritis and mortality syndrome. Poult Sci.

[35] Pluske JR, Turpin DL, Kim JC (2018). Gastrointestinal tract (gut) health in the young pig. Anim Nutr.

[36] Chen F, Wang H, Chen J, Liu Y, Wen W, Li Y (2020). Lactobacillus delbrueckii ameliorates intestinal integrity and antioxidant ability in weaned piglets after a lipopolysaccharide challenge. Oxid Med Cell Longev.

[37] Song Z, Tong G, Xiao K, le Jiao F, Ke Y, Hu C (2016). L-cysteine protects intestinal integrity, attenuates intestinal inflammation and oxidant stress, and modulates NF-kappaB and Nrf2 pathways in weaned piglets after LPS challenge. Innate Immun.

[38] Guerra-Ordaz AA, Gonzalez-Ortiz G, La Ragione RM, Woodward MJ, Collins JW, Perez JF (2014). Lactulose and Lactobacillus plantarum, a potential complementary synbiotic to control postweaning colibacillosis in piglets. Appl Environ Microbiol.

[39] Xiao D, Tang Z, Yin Y, Zhang B, Hu X, Feng Z (2013). Effects of dietary administering chitosan on growth performance, jejunal morphology, jejunal mucosal sIgA, occludin, claudin-1 and TLR4 expression in weaned piglets challenged by enterotoxigenic Escherichia coli. Int Immunopharmacol.

[40] Lee SI, Kang KS (2019). N-acetylcysteine modulates lipopolysaccharide-induced intestinal dysfunction. Sci Rep.

[41] Wang H, Liu Y, Shi H, Wang X, Zhu H, Pi D (2017). Aspartate attenuates intestinal injury and inhibits TLR4 and NODs/NF-kappaB and p38 signaling in weaned pigs after LPS challenge. Eur J Nutr.

[42] Xiao K, Jiao L, Cao S, Song Z, Hu C, Han X (2016). Whey protein concentrate enhances intestinal integrity and influences transforming growth factor-beta1 and mitogen-activated protein kinase signalling pathways in piglets after lipopolysaccharide challenge. Br J Nutr.

[43] Hu R, He Z, Liu M, Tan J, Zhang H, Hou DX (2020). Dietary protocatechuic acid ameliorates inflammation and up-regulates intestinal tight junction proteins by modulating gut microbiota in LPS-challenged piglets. J Anim Sci Biotechnol.

[44] Chen F, Chen J, Chen Q, Yang L, Yin J, Li Y, et al. *Lactobacillus delbrueckii* protected intestinal integrity, alleviated intestinal oxidative damage, and activated toll-like receptor-Bruton's tyrosine kinase-nuclear factor erythroid 2-related factor 2 pathway in weaned piglets challenged with lipopolysaccharide. Antioxidants (Basel). 2021;10(3):468. 10.3390/antiox10030468. 10.3390/antiox10030468 PMC8002333 33809627

[45] Schumann C (2002). Medical, nutritional and technological properties of lactulose. An update Eur J Nutr.

[46] Krueger M, Schroedl W, Isik W, Lange W, Hagemann L (2002). Effects of lactulose on the intestinal microflora of periparturient sows and their piglets. Eur J Nutr.

[47] Wan MLY, Ling KH, El-Nezami H, Wang MF (2019). Influence of functional food components on gut health. Crit Rev Food Sci Nutr.

[48] Ohland CL, Macnaughton WK (2010). Probiotic bacteria and intestinal epithelial barrier function. Am J Physiol Gastrointest Liver Physiol.

[49] Wang Y, Xie Q, Zhang Y, Ma W, Ning K, Xiang JY (2020). Combination of probiotics with different functions alleviate DSS-induced colitis by regulating intestinal microbiota, IL-10, and barrier function. Appl Microbiol Biotechnol.

[50] Xie XL, He JT, Wang ZT, Xiao HQ, Zhou WT, Du SH (2018). Lactulose attenuates METH-induced neurotoxicity by alleviating the impaired autophagy, stabilizing the perturbed antioxidant system and suppressing apoptosis in rat striatum. Toxicol Lett.

[51] Xing SC, Huang CB, Mi JD, Wu YB, Liao XD (2019). Bacillus coagulans R11 maintained intestinal villus health and decreased intestinal injury in lead-exposed mice by regulating the intestinal microbiota and influenced the function of faecal microRNAs. Environ Pollut.

