# Supplementary material for: The synbiotic mixture of lactulose and Bacillus coagulans protects intestinal barrier dysfunction and apoptosis in weaned piglets challenged with lipopolysaccharide
Source: J Anim Sci Biotechnol. 2023 Jun 11;14:80. doi: 10.1186/s40104-023-00882-9 (PMC10257848; doi:10.1186/s40104-023-00882-9)
Supplement: Supplementary file 1 — Additional file 1: Fig. S1. Flow chart of this study. Fig. S2. Effects of synbiotic mixture of lactulose and Bacillus coagulans on the diarrhea in piglets during the feeding trial. Table S1. Ingredient composition and nutrient contents of the basal experimental diet. Table S2. List of primers used in this study. [file 40104_2023_882_MOESM1_ESM.docx]

**
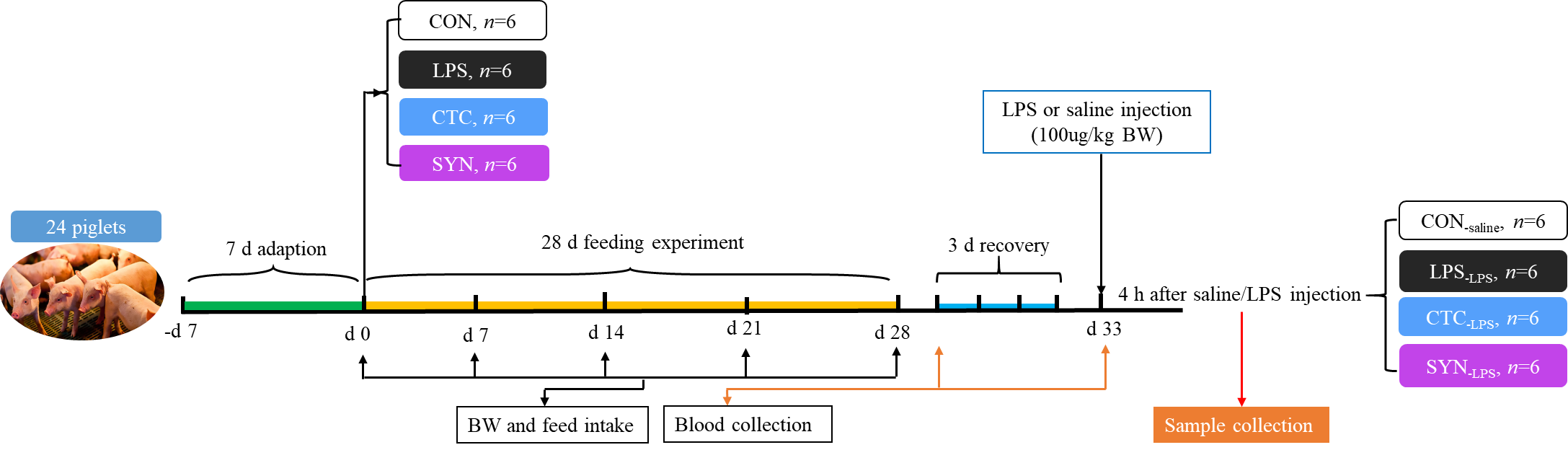
**

**Fig. S1** Flow chart of this study

**
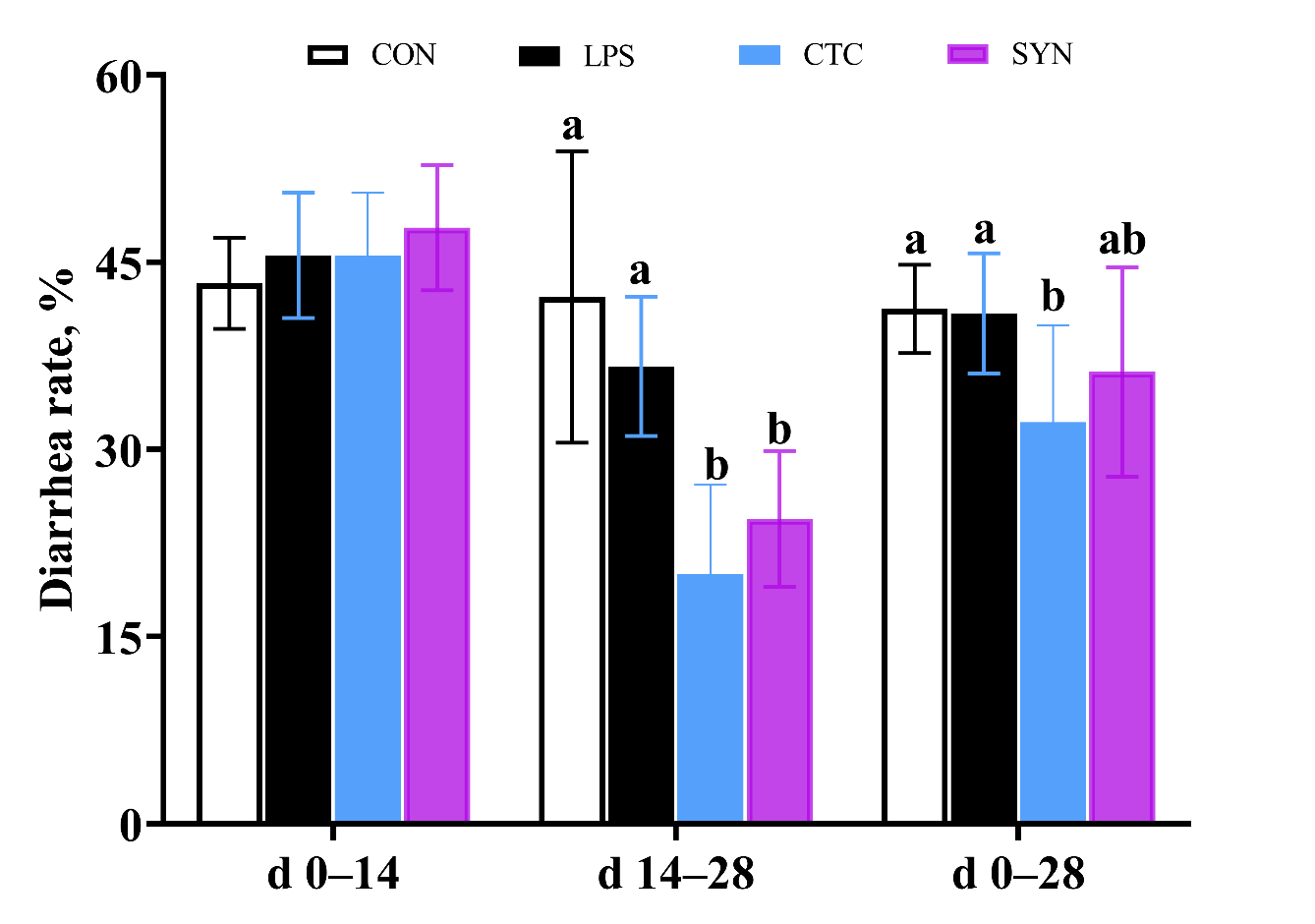
**

**Fig. S2** Effects of synbiotic mixture of lactulose and *Bacillus coagulans* on the diarrhea in piglets during the feeding trial

**Table S1** Ingredient composition and nutrient contents of the basal experimental diet

| **Item** | **Content** |
| --- | --- |
| Corn | 51.07 |
| Soybean meal | 15.00 |
| Extruded soybean | 8.58 |
| Whey power | 6.00 |
| Wheat bran | 5.00 |
| Fish meal | 5.00 |
| Soybean oil | 2.00 |
| Premix^1^ | 1.00 |
| Sugar | 2.00 |
| Glucose | 2.00 |
| CaHPO_4_ | 0.60 |
| Limestone | 0.55 |
| NaCl | 0.30 |
| TiO_2_ | 0.30 |
| *L*-Lysine HCl | 0.23 |
| *L*-Threonine | 0.15 |
| *DL*-Methionine | 0.10 |
| *L*-Tryptophan | 0.07 |
| Choline chloride | 0.05 |
| Total | 100.00 |
| Calculated nutrient levels % | |
| Metabolite energy (ME), kcal/kg | 3,336.52 |
| Standardized ileal digestibility (SID) AA | |
| Lys | 1.29% |
| Met | 0.41% |
| Trp | 0.23% |
| Thr | 0.74% |
| Analyzed nutrient levels, % | |
| Gross energy, kcal/kg | 4,177.82 |
| Crude protein | 18.99 |
| Ether extract | 6.64 |
| Crude fiber | 2.46 |
| Total phosphorus | 0.74 |
| Calcium | 0.75 |

^1^The premix provided the following per kg of diets: Fe (C_4_H_8_FeN_2_O_4_) 105 mg, Cu (CuSO_4_·5H_2_O) 125 mg, Zn (ZnSO_4_·H_2_O) 22 mg, Mn (MnSO_4_·H_2_O) 20 mg, I [Ca(IO_3_)_2_] 0.1 mg, VA 124.5 mg, VD_3_ 23.8 mg, VE 1044.4 mg, VK_3_ 34.4 mg, VB_1_ 17.6 mg, VB_2_ 89.4 mg, VB_6_ 14.0 mg, pantothenic acid 237.3 mg, nicotinic acid 430.5 mg, folic acid 19.4 mg, biotin 3.2 mg

**Table S2** List of primers used in this study

| **Gene^1^** | **Primer sequences (5’→3’)^2^** | **Size, bp** | **A_T_^3^, °C** | **Reference** |
| --- | --- | --- | --- | --- |
| *ZO-1* | F: CAGAGACCAAGAGCCGTCC | 105 | 60 | [1] |
|  | R: TGCTTCAAGACATGGTTGGC |  |  |  |
| *ZO-2* | F: GCAGAGACAACCCCCACTTT | 117 | 55.8 | [2] |
|  | R: CGTTAACCATGACCACCCGA |  |  |  |
| *OCLN* | F: TCAGGTGCACCCTCCAGATT | 118 | 60 | [1] |
|  | R: AGGAGGTGGACTTTCAAGAGG |  |  |  |
| *CLDN-1* | F: TTCTGGGAGGTGCCCTACTT | 74 | 60 | [3] |
|  | R: TGGATAGGGCCTTGGTGTTG |  |  |  |
| *CLDN-2* | F: CTCGTTGGCCTGTATCATCACC | 181 | 63.1 | [2] |
|  | R: CAGGGGGGAGTAGAAGTCCC |  |  |  |
| *CLDN-3* | F: AACACCATCATCCGGGACTTC | 117 | 61.2 | [2] |
|  | R: CGCGGAGTAGAGGATCTTGG |  |  |  |
| *CLDN-4* | F: GTATCATCCTGGCCGTGCTA | 82 | 60 | This study |
|  | R: TTGGCGCTCTCATCATCCA |  |  |  |
| *CLDN-5* | F: CTCTGCTGGTTCGCCAACA | 75 | 58.7 | [2] |
|  | R: CAGCTCGTACTTCTGCGACATG |  |  |  |
| β-actin | F: GGACTTCGAGCAGGAGATGG | 233 | 60 | [4] |
|  | R: GCACCGTGTTGGCGTAGAGG |  |  |  |

^1^Gene symbols are used for target. *ZO-1*= zonula occluden 1, *ZO-2*= zonula cooluden 2, *OCLN*= occludin, *CLDN-2*= claudin 2, *CLDN-3*= claudin 3, *CLDN-4*= claudin 4, *CLDN-5*= claudin 5

^2^F=forward primer; R=reverse primer

^3^A_T_=annealing temperature

**Reference**

1. Zhang Y, Zheng P, Yu B, He J, Yu J, Mao XB, et al. Dietary spray-dried chicken plasma improves intestinal barrier function and modulates immune status in weaning piglets. J Anim Sci. 2016;94(1):173–84. doi: 10.2527/jas.2015-9530.

2. Alizadeh A, Braber S, Akbari P, Garssen J, Fink-Gremmels J. Deoxynivalenol impairs weight gain and affects markers of gut health after low-Dose, short-Term exposure of growing pigs. Toxins (Basel). 2015;7(6):2071–95. doi: 10.3390/toxins7062071.

3. Qi KK, Wu J, Deng B, Li YM, Xu ZW. PEGylated porcine glucagon-like peptide-2 improved the intestinal digestive function and prevented inflammation of weaning piglets challenged with LPS. Animal. 2015;9(9):1481–9. doi: 10.1017/S1751731115000749.

4. Li G, Yao W, Jiang H. Short-chain fatty acids enhance adipocyte differentiation in the stromal vascular fraction of porcine adipose tissue. J Nutr. 2014;144(12):1887–95. doi: 10.3945/jn.114.198531.
